# Closing the gap between training needs and training provision in addiction medicine

**DOI:** 10.1192/bji.2019.27

**Published:** 2020-05

**Authors:** Sidharth Arya, Mirjana Delic, Blanca Iciar Indave Ruiz, Jan Klimas, Duccio Papanti, Anton Stepanov, Victoria Cock, Dzmitry Krupchanka

**Affiliations:** 1 MD, Assistant Professor, State Drug Dependence Treatment Centre, Institute of Mental Health, Pt BDS University of Health Sciences, India; 2 PhD, Psychiatrist, Center for Treatment of Drug Addiction, University Psychiatric Hospital Ljubljana, Slovenia; 3 PhD, Research Assistant, National Center for Epidemiology, Carlos III Institute of Health, Spain; 4 PhD, Senior Postdoctoral Fellow, British Columbia Centre on Substance Use, University of British Columbia, Canada; 5 MD, Psychiatrist, Department of Mental Health, Integrated University Healthcare Company of Udine (ASUIUD), Italy; 6 MD, Head of Rehabilitation Department, Gomel Regional Narcological Dispensary, Belarus; 7 FAChAM (RACP), Consultant Addiction Medicine Specialist, Drug and Alcohol Services of South Australia, South Australia; 8 PhD, Medical Officer, Management of Substance Abuse Unit, Department of Mental Health and Substance Abuse, World Health Organization, Geneva, Switzerland. Email: dmitry.krupchenko@gmail.com

**Keywords:** Education and training, alcohol disorders, drugs of dependence disorders

## Abstract

Substance use disorders pose a significant global social and economic burden. Although effective interventions exist, treatment coverage remains limited. The lack of an adequately trained workforce is one of the prominent reasons. Recent initiatives have been taken worldwide to improve training, but further efforts are required to build curricula that are internationally applicable. We believe that the training needs of professionals in the area have not yet been explored in sufficient detail. We propose that a peer-led survey to assess those needs, using a standardised structured tool, would help to overcome this deficiency. The findings from such a survey could be used to develop a core set of competencies which is sufficiently flexible in its implementation to address the specific needs of the wide range of professionals working in addiction medicine worldwide.

Substance use and substance use disorders (SUDs) are major contributory factors to global morbidity and mortality. According to recent reports, 43% (2.3 billion) of the global adult population have consumed alcohol during past 12 months.^1^ A 2016 survey found that more than 5% of people (283 million) had alcohol use disorders; about 6% (275 million) had used other drugs at least once and about 0.6% (31 million) had drug use disorders.^2^ Annually, about 3 million deaths are attributable to alcohol use and 450 000 deaths are attributable to other forms of drug use. A systematic analysis of the global burden of disease (1990–2016) concluded that 4.2% and 1.3% of all disability-adjusted life years (DALYs) were attributable to alcohol and drug use respectively.^3^ Such trends have been of increasing concern to public health services. Strengthening the workforce in the field of addiction medicine would contribute to addressing these challenges.

Effective pharmacological and psychosocial strategies exist for treatment of SUDs. Although improving access to treatment requires multifaceted approaches at all levels of the system, increasing the size of an adequately trained workforce can potentially improve treatment outcomes. Despite SUDs being a common public health problem, there seems to be a reluctance among general practitioners, physicians and psychiatrists to treat and manage these conditions.^4^ In addition to cultural, political and legal impediments, stigma and a self-perceived lack of competence in handling SUDs have been cited as important factors that contribute to negative professional attitudes, such as having a low regard for persons with SUDs and a reluctance to work with them.

## Current training in addiction medicine

### Worldwide

A review of the organisation and effectiveness of addiction medicine training across the globe highlighted a lack of standardised training programmes at both graduate and postgraduate levels of medical education, with the exception of few psychiatry and family medicine courses.^5^ There are several barriers to developing addiction training in the medical education curriculum. These include the limited availability of curricular time, poor interdepartmental coordination, insufficient faculty members qualified to teach addiction medicine and a lack of addiction treatment facilities that can be used as education sites for clinical experience.^5^

The World Health Organization (WHO) ATLAS-SU survey on resources for the prevention and treatment of SUDs, conducted in 162 countries,^6^ highlighted the deficiencies in training programmes on SUDs globally. Almost one-third of all countries surveyed reported that they had no training programmes for their workforce in the management of SUDs; that figure increased to 60% of low-income countries. Worldwide, the greatest availability of postgraduate training exists for psychiatrists (52% of countries) and other doctors (49%). The lowest availability of courses for those specialising in addiction medicine were for counsellors (23%) and community health workers (19%).

The WHO, in laying out its global strategy on human resources for health, has emphasised the importance of building the capacity and capability of the workforce in order to achieve a range of sustainable development goals.^7^ Over the past decade, efforts have been made to create a specialist training pathway in the field of addiction medicine. Numerous training programmes have been initiated in high-income countries.^8^ For example, countries such as Canada, The Netherlands, the USA and Australia have initiated Fellowships in Addiction Medicine, and some (Canada, USA) have extended these schemes to non-physicians. Most low- and middle-income countries do not have such fellowships and access to quality education at the undergraduate/medical school level remains a problem. It is beyond doubt that programmes aimed at increasing the number of professionals specialising in this field will improve the overall quality of care for SUDs, and the lack of such programmes in low-resource settings, which have a higher burden of SUDs and poorer access to treatment, remains a problem.

### Low- and middle-income countries

Traditionally, training in SUDs in the majority of low- and middle-income countries has followed an *ad libitum* approach. Instead of having a well laid-out plan for training, the provision of care (and related training in the field) has often been provided on *ad hoc* basis and is often inadequate. Such programmes are usually short- term, focus on a narrow set of aims and are often planned with limited consideration of future training needs. In our opinion, an approach is required which is not as resource intensive as specialised training, but which targets professionals' needs by taking into account their views and providing clear goals and flexibility in implementation.

### Course content

Although there is a substantial literature highlighting the deficiencies in training courses on SUDs for undergraduates and postgraduates, the format and content of professional training courses has not been fully characterised. In particular, there is little information available about the way in which low-income countries could fulfil the training needs of medical and other professionals. As a result, it is difficult to make recommendations on the basis of existing evidence. For example, a 2015 review of the organisation and effectiveness of addiction medicine training across the world could find only nine studies in the previous 15 years that provided information about training needs.^5^ Five of these studies were from the USA. They evaluated faculty perceptions about existing services available for residents in addiction training. One German study assessed the services available for undergraduates. Only one study aimed to develop a curriculum in addiction medicine for healthcare professionals and explored training needs from their perspective. This study proposed a number of core competencies, including screening, assessment and diagnosis, management and treatment, and management and referral of medical comorbidities.^9^

## A call for action: systematic training-needs assessment

In the absence of scientific evidence, recommendations have been made by international scholars that highlight a core set of competencies to be covered at undergraduate and postgraduate levels, as well in continued medical education.^10^ Although relevant, these recommendations cannot be assumed to be representative of the competencies required in day-to-day practice. As members of the international Network of Early Career Professionals in Addiction Medicine (NECPAM), we call for a web-based survey to enable systematic assessment of the training needs of the professionals working in addiction medicine, at various educational levels and in a wide variety of countries. This proposed assessment will be peer-led, it will use structured, validated instruments to gather data and it will focus in particularly on early career professionals working in addiction medicine across many countries.

Professionals who manage SUDs include psychiatrists, specialist physicians, general practitioners, nurses, psychologists, social workers and counsellors. Each has a specific range of training needs. Furthermore, these health professionals are expected to provide different aspects of SUD service requirements. There are significant sociodemographic, cultural and political characteristics that influence the management of SUDs in different regions. As discussed, there are vast differences in the training in SUD management that is provided in different parts of the world, with specialist courses in some regions but a complete lack of basic health workers in all medical fields in others. The globally distributed training-needs survey would help highlight basic deficits in skills and knowledge. Those needs could subsequently be addressed by the development of a peer-led learning model that yields itself to being adapted for use in any region.

Apart from focusing on training needs among a wide variety of professions, the emphasis of such a survey must also be on those professionals who are at an early career stage. These are the individuals who are likely to be the leaders and stakeholders in the field of addiction medicine in the future. Therefore, addressing the issues most pertinent to them will empower and enhance their capabilities.^11^ Investing in early career professionals will serve as a timely replacement for an ageing workforce in addiction medicine.^12^

## Conclusions

Worldwide, addiction medicine training programmes differ significantly in their content and duration (from just a few weeks to several years). Because of such heterogeneity, the clinical skills that are taught by such programmes vary in quality and are usually insufficient. As early career professionals from different countries we feel that better training is required to handle SUD prevention and management. There should be an enhanced focus on the identification of the specific training needs of the early career health professionals engaged in providing SUD services.

We are advocating that a core set of competencies be developed and that particular attention be paid to the views of those already working in the field. The recommendations should be sufficiently flexible in their implementation to be sensitive to a variety of specific needs, depending on the culture and characteristics of the country in which those professionals are working. There should be a structured set of objectives with appropriate flexibility in terms of their implementation. To achieve our goal, we suggest that professionals are asked about what they really need to know. As a starting point, we aim to assess the training needs of addiction professionals worldwide. We believe that combining these evidence-based findings on perceived training needs with a modified and flexible educational programme for healthcare workers would help to make existing programmes more relevant, effective and acceptable to treatment providers and patients alike.
